# Aseptic Operative Technic

**Published:** 1907-10

**Authors:** John Egerton Cannaday

**Affiliations:** Sheltering Arms Hospital, Surgeon-in-Chargc, Hansford, W. Va.


### Aseptic Operative Technic.

By JOHN EGERTON CANNADAY, M. D.,
Sheltering Arms Hospital, Surgeon-in-Charge, Hansford, W. Va.

*(Author's Abstract.)*

THE author points out the advantages of a simple, logical and consistent technic. He says the surgeon must possess the instinct of cleanliness. The preoperative treatment is sketched in its principle essentials. The gastrointestinal tract is emptied of the bulk of its contents. The various details that go so far toward excellence of technic are described. Methods of skin cleansing are described and the more desirable antiseptic solutions are discussed. The proper method of putting on gloves so that the outside of the glove will not touch the bare skin is described and illustrated. Many operators defeat the end aimed at by lack of attention to these details. Especial attention should be devoted to the cleansing of and care of the hands and the keeping the skin in the pink of condition. The advantages of the layer sutures of absorbable material are pointed out. Subcutaneous suturing lessens wound infection and gives a neat approximation. Irrigation of the peritoneal cavity generally speaking is condemned.

In closing the author's conclusions are as follows: "That bichloride of mercury as ordinarily employed is useless and engenders a false sense of security.

That the bugaboos of prolonged scrubbing of the hands and arms, with rough brushes and reckless use of strong bichloride solutions favor rather than diminish the chances of infection in the long run.

That the iodine solution is comparatively nontoxic and highly antiseptic. Laboratory experiments have conclusively proved that as a germicide a 1:500 solution of iodine will do in five minutes what a 1:1000 solution of bichloride will take half an hour in accomplishing.

That persistent care of the hands and the wearing of gloves is of prime importance to protect the patient from the operator, and to protect the operator from the patient. The objections are slight as compared to many advantages accruing from their use. Face masks are advantageous in the prevention of wound infection. Longsleeved gowns are indicated for the sake of consistency in technic.

That irrigation and attempted drainage of the abdominal cavity are as a rule inadequate and harmful.

That whenever possible unabsorbable buried suture material should be avoided. That the evils of drainage are many and the indications for its use are few. When in doubt do not drain.

The Kelly's method of starvation diet to prevent the formation of scybalous masses that might tear newly united perineal tissues, and of locking the bowels for from ten days to two weeks to allow ample time for healing after plastic operations on rectum and perineum has many advantages."

